# Trametes robiniophila represses angiogenesis and tumor growth of lung cancer via strengthening let-7d-5p and targeting NAP1L1

**DOI:** 10.1080/21655979.2021.2012619

**Published:** 2022-03-01

**Authors:** HuiZhu Gan, XinXin Xu, YinYin Bai

**Affiliations:** Department of Hematology and Oncology, China-Japan Union Hospital of Jilin University, Changchun City, JiLin Province, China

**Keywords:** Trametes robiniophila, let-7d-5p, NAP1L1, angiogenesis, lung cancer

## Abstract

Trametes robiniophila (Huaier) is available to refrain lung cancer (LC) cell progression, but its impact and mechanism on angiogenesis of LC are not proved. The study was to explore the potential mechanism of Huaier repressing angiogenesis and tumor growth in LC via strengthening let-7d-5p and targeting NAP1L1. Let-7d-5p and NAP1L1 expression was detected in LC tissues and cells (A549). Pretreatment of A549 cells was with Huaier. Transfection of changed let-7d-5p and NAP1L1 was to A549 cells to uncover their roles in LC cell progression with angiogenesis. Evaluation of the impact of let-7d-5p on angiogenesis in LC was *in vitro* in a mouse xenograft model. Identification of the targeting of let-7d-5p with NAP1L1 was clarified. The results clarified reduced let-7d-5p but elevated NAP1L1 were manifested in LC. Huaier restrained angiogenesis and tumor growth of LC *in vivo* and *in vitro*; Augmented let-7d-5p or declined NAP1L1 motivated the therapy of Huaier on LC; Let-7d-5p negatively modulated NAP1L1; Elevated NAP1L1 reversed the influence of enhancive let-7d-5p. These results strongly suggest that Huaier represses angiogenesis and tumor growth in LC via strengthening let-7d-5p and targeting NAP1L1. Huaier/let-7d-5p/NAP1L1 axis is supposed to be a promising target for the treatment of angiogenesis and tumor growth in LC via elevated let-7d-5p and targeted NAP1L1.

## 1 Introduction

Lung cancer (LC) is a familiar and fatal malignancy in most countries, with the surprising clinical fatality [1]. Inchoate detection of LC can be achieved via analysis of respiratory tissue samples or application of biomarkers in peripheral biological fluids like blood and urine. Nevertheless, no detailed study has been clarified on the pathogenesis of LC, seriously restricting the clinical treatment and effective prevention of the disease. Hence, a crying need is pointed out for discovering brand-new biomarkers and molecular mechanisms to mitigate LC treatment.

Traditional Chinese medicine (TCM) has been extensively applicated in the prevention and treatment of malignancies in China for a long period [2]. Owing to its abundant natural resources, excellent efficacy, few toxicities and diverse chemical ingredients, it has been welcomed via people. Huaier, also known as Trametes robiniophila Murr, is a sandy beige mushroom, which has been used in traditional Chinese medicine (TCM) with a history of more than 1600 years. Huaier aqueous extract and Huaier granule, with the tradename of Jinke, are the most commonly used types clinically. According to the analysis of high performance liquid chromatography (HPLC) and sulfate polyacrylamide gel electrophoresis (SDS-PAGE), the most effective component of Huaier was identified as proteoglycan, which included 41.53% polysaccharide, 12.93% amino acid and 8.72% water [3]. Polysaccharides were identified as possible key components in Huaier [4–6]. The anti-tumor achievements of Huaier have been employed in the complementary therapy of all kinds of cancers, like cervical [7], breast [8,9], kidney [10], hepatocellular [11,12] and non-small lung cell carcinomas [13]. Nevertheless, the downstream modulation of mechanism of Huaier is ill-informed.

**Figure 1.**
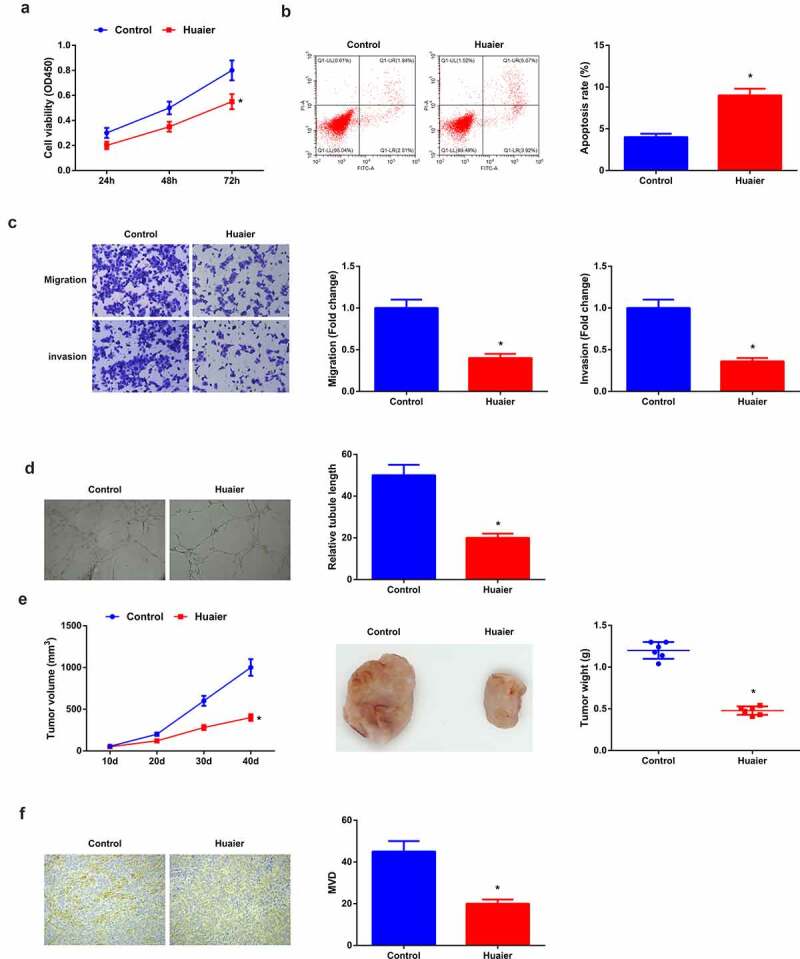
**Huaier constrains LC angiogenesis and tumor growth *in vivo* and *in vitro.*** (a) CCK-8 detection of cell proliferation; (b) Flow cytometry detection of cell apoptosis; (c) Transwell detection of cell migration and invasion; (d) Matrigel tube formation test of the angiogenesis ability (200 ×); (e) Tumor volume and mass (*n* = 6); (f) Immunohistochemical characterization of CD31 protein and calculation of angiogenesis (*n* = 6); Expression of the values was as mean ± SD (*N* = 3 in cell experiments). In the Control and the Huaier groups. * vs. the Control, *P* < 0.05. **Attached** Figure 1 **Down-regulation of let-7 family expression is presented in LC** A. qRT-PCR to detect let-7 family expression level in LC; B. qRT-PCR to detect let-7d-5p expression levels in immortalized human lung cell line Beas-2B and LC cell lines (A549, H460, H1299, H1650 and H1975). The values were expressed as mean ± SD (cell experiments were repeated 3 times independently). One-way ANOVA was applied to calculate the significance of each group. The variance was corrected by Tukey test. * vs. the Normal group, *P* < 0.05. 0.05.

MicroRNAs (miRNAs), a group of little non-coding RNAs, are available to repress target genes expression or mediate their degradation [14]. Abnormal miRNAs are implicated with various cellular pathways and modulate the proliferation and metastasis of all kinds of tumors [15]. Studies have shown that elevated let-7a-5p can induce apoptosis of LC cells [16]; Let-7b-5p is downregulated in hepatocellular carcinoma, and elevated one can repress cell metastasis and EMT progression [17]. The expression of let-7 c-5p in breast cancer is decreased, and the overexpression of let-7 c-5p can apparently depress cell proliferation but induce cell apoptosis [18]. Let-7d-5p is reduced in colorectal cancer, and elevation of let-7d-5p can inhibit the development and metastasis of colorectal cancer [19]. Overexpression of let-7e-5p restrains the proliferation, migration and invasion of head and neck squamous cell carcinoma cells [20]. Therefore, it was suspected that let-7 family is related to cancer development. Relevant preliminary experiments were conducted and the expression level of let-7 family (let-7a-5p, let-7b-5p, let-7 c-5p, let-7d-5p, let-7e-5p, let-7 f-5p, let-7 g-5p, let-7i-5p) was detected in LC via qRT-PCR. The results found that the expression of let-7 family was reduced in LC, and the expression level of let-7d-5p changed most apparently. Therefore, let-7d-5p was chosen as the focus of the study (Attached Figure 1(a)). However, the role of let-7d-5p in the progression of LC is still unknown. In addition, it was also found that the expression level of let-7d-5p was decreased in LC cell lines (A549, H460, H1299, H1650 and H1975), and the expression level of let-7d-5p changed most clearly in A549 cells. Therefore, A549 cells were selected for the follow-up experiments (Attached Figure 1(b)).

**Figure 2.**
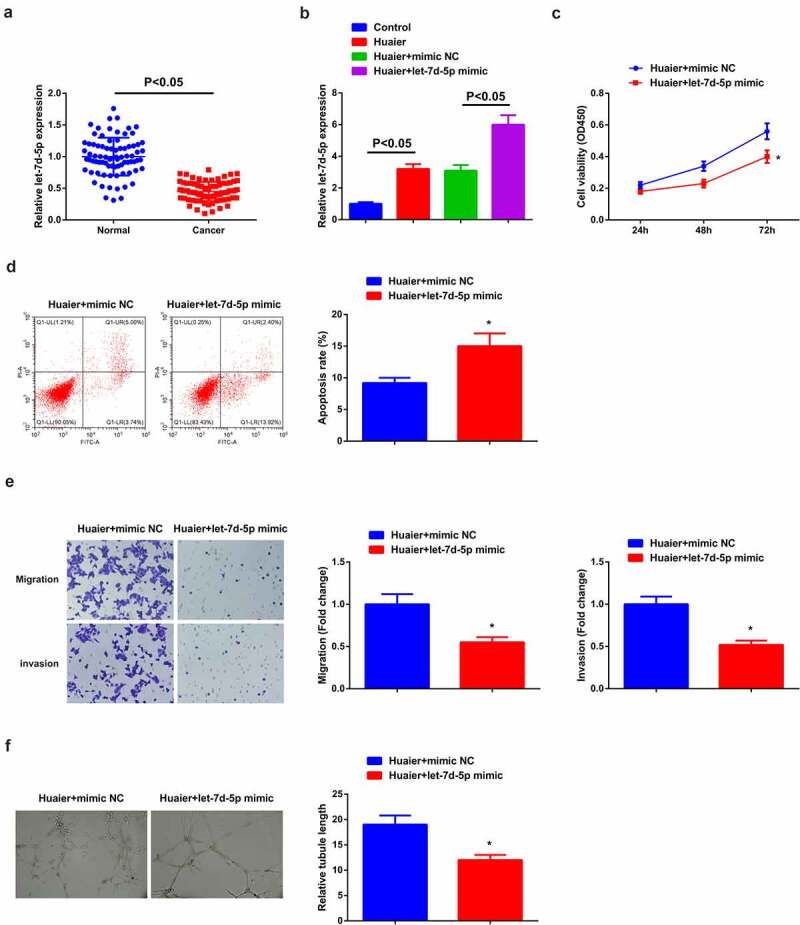
**Restrained let-7d-5p is in LC; elevated let-7d-5p accelerates Huaier’s therapy on LC.** (a) RT-qPCR detection of let-7d-5p in clinical samples; (b) RT-qPCR verification of the successful transfection; (c) CCK-8 detection of cell proliferation; (d) Flow cytometry detection of cell apoptosis; (e) Transwell detection of cell migration and invasion; (f) Matrigel tube formation test of the angiogenesis ability (200 ×); (C–F) after elevated let-7d-5p. Expression of the values was as mean ± SD (*N* = 3). * vs. the Huaier + mimic NC, *P* < 0.05. **Attached** Figure 2 **Huaier represses the proliferation, migration, invasion and angiogenesis of H460 cells, but promotes apoptosis.** (a) CCK-8 to detect the proliferation of cells in the Control and the Huaier groups; (B)Cell apoptosis in the Control and the Huaier groups detected by flow cytometry; (c) Transwell to detect the migration and invasion of cells in the Control and the Huaier groups; (d) Matrigel tube formation assay to detect angiogenesis in the Control and the Huaier groups (200 ×); The values were expressed as mean ± SD (cell experiments were repeated 3 times independently). One-way ANOVA was applied to calculate the significance of each group. The variance was corrected by Tukey test. * vs. the Control group, *P* < 0.05.

**Figure 3.**
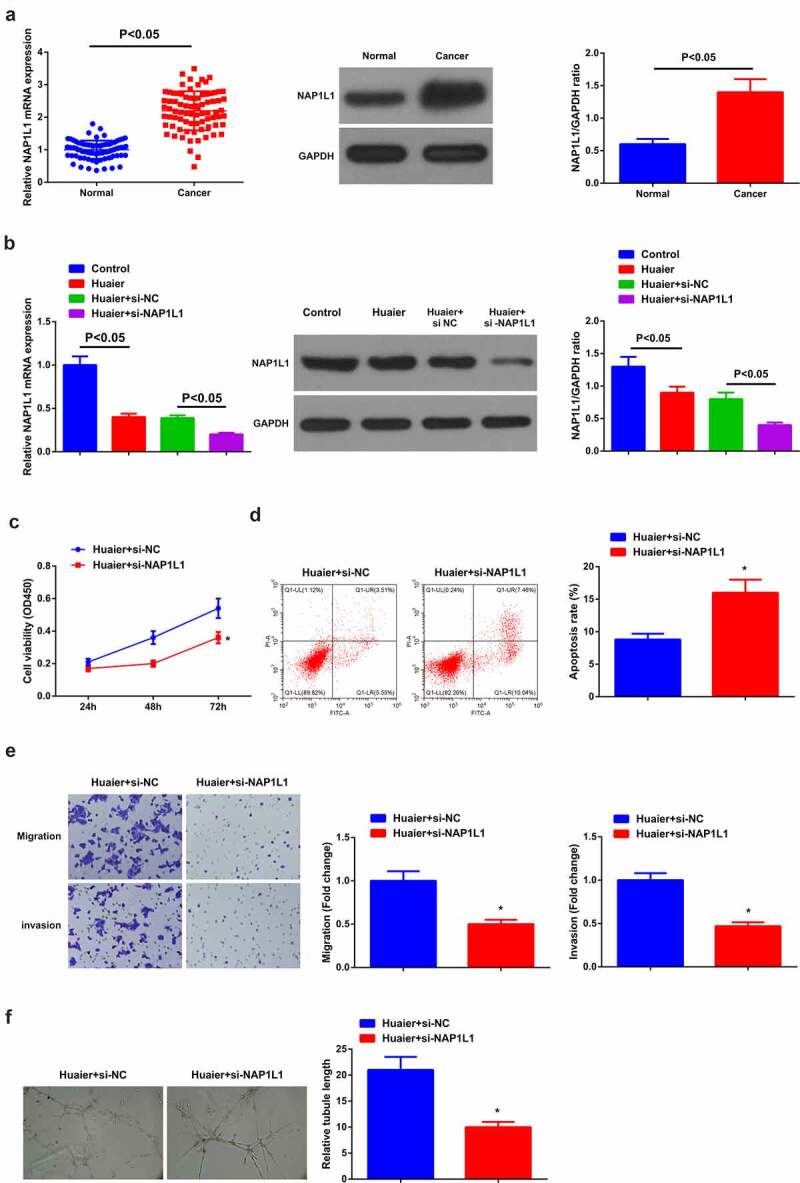
**Elevated NAP1L1 is in LC; constrained NAP1L1 expedites Huaier’s therapy on LC.** (a) RT-qPCR and Western blot detection of NAP1L1 in clinical samples; (b) RT-qPCR and Western blot verification of the successful transfection; (c) CCK-8 detection of cell proliferation; (d) Flow cytometry detection of cell apoptosis; (e) Transwell detection of cell migration and invasion; (f) Matrigel tube formation test of the angiogenesis ability (200 ×); C-F, after depressive NAP1L1. Expression of the values was as mean ± SD (*N* = 3). * vs. the Huaier + si-NC, *P* < 0.05.

**Figure 4.**
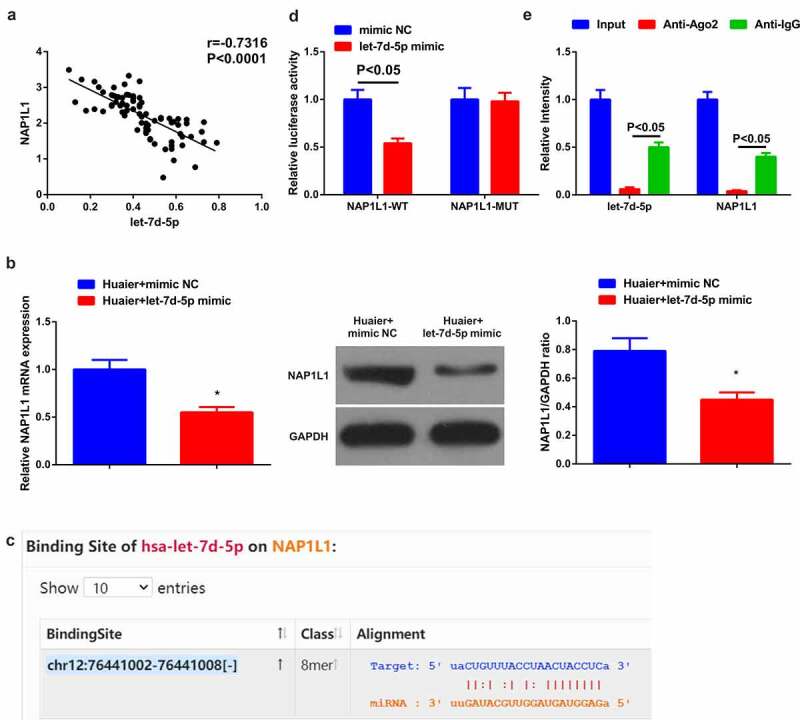
**Let-7d-5p targets NAP1L1.** (a) Correlation analysis of let-7d-5p and NAP1L1 in tissues; (b) NAP1L1 after elevated let-7d-5p detected via RT-qPCR and Western blot; (c) Starbase database prediction of the binding site of let-7d-5p to NAP1L1; (d) The luciferase activity assay verification of the targeting of the two; Expression of the values was as mean ± SD (*N* = 3). * vs. the Huaier + mimic NC, *P* < 0.05.

**Figure 5.**
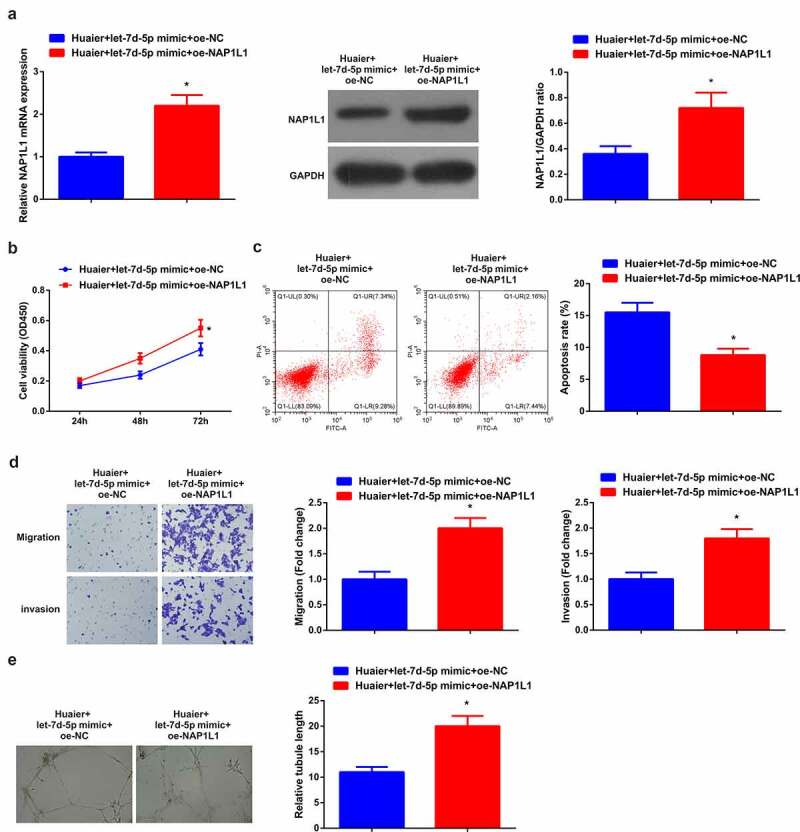
**Enhanced NAP1L1 turns around the impact of elevated let-7d-5p.** (a) RT-qPCR and Western blot detection of NAP1L1; (b) CCK-8 detection of cell proliferation; (c) Flow cytometry detection of cell apoptosis; (d) Transwell detection of cell migration and invasion; (e). Matrigel tube formation test of the angiogenesis ability (200 ×); Expression of the values was as mean ± SD (*N* = 3). * vs. the Huaier + let-7d-5p mimic + oe-NC, *P* < 0.05.

NAP1L1, the human counterpart of the yeast NAP-1 protein, and a kind of histone-binding factor involved in cumulative nucleosome formation, has been testified to be a potential tumor promoter and participates in the pathogenesis of a variety of tumors, including colorectal cancer, renal carcinoma and pancreatic neuroendocrine neoplasms [21–23]. In the meantime, a study has manifested the augment of NAP1L1 in hepatocellular carcinoma (HCC) with motivation of the growth and metastasis of cells [24]. However, NAP1L1 has been poorly studied in LC.

Inspired via these explorations, this study was used to explore the repression of angiogenesis and tumor growth in LC via elevated let-7d-5p and targeting NAP1L1 via Huaier.

## 2 Materials and methods

### 2.1 Clinical sample collection

This study was approved by the Research Ethics Committee of China–Japan Union Hospital of Jilin University. Collection of 80 pairs of LC patients and their corresponding normal controls was from the Department of Cardiothoracic Surgery in China–Japan Union Hospital of Jilin University. Written informed consent waere obtained from all the patients.

### 2.2 Cell culture

The culture of human LC cell line A549 and human umbilical vein endothelial cells (HUVECs) (Beijing Zhongyuan Ltd., China) was in Dulbecco’s Modified Eagle’s medium (DMEM, Gibco) with 10% fetal bovine serum (FBS, Gibco). Electuary ointment of Huaier (Gaitianli, Jiangsu, China) was dissolved in phosphate-buffered saline (PBS), and the filtration was conducted via 0.22 μm filter to gain 100 mg/mL original solution, which was then diluted into 4 mg/mL working solution before use. In the Huaier group, the incubation of A549 cells was with Huaier, while that of the control was with the same amount of elevated glucose complete DMEM [25].

### 2.3 Cell transfection

Construction of let-7d-5p mimic, mimic NC, si/oe-NAP1L1 and si/oe-NC was in line with the instructions of Lipofectamine™RNAiMAX (Invitrogen, Carlsbad, CA, USA). On the grounds of the manufacturer’s protocol, transfection of Huaier-treated A549 cells was with Lipofectamine RNAiMAX.

### 2.4 Cell counting kit (CCK)-8

Seeding of A549 cells (3000 cells/well) was in triplicate into 96-well plates. After culture, the incubation of 10 μL CCK-8 solution (Beyotime, Shanghai, China) was to each well. Measurement of the optical density of each well was at 450 nm via a microplate reader (Bio-Rad, Hercules, CA, USA) [26].

### 2.5 Flow cytometry

The seeding of the cells (1 × 10^6^ cells/well) was in 6-well plates and harvest was after transfection. Application of Annexin V/propidium iodide kit (KeyGEN, Nanjing, China) was for apoptosis assay, with that of FACScalibur flow cytometry (BD Biosciences, San Jose, CA, USA) for examination of the percentage of apoptotic cells.

### 2.6 Transwell

After transfection, starvation of the cells was in a serum-free medium with which adjustment of the density was to 3 × 10^4^ cells/mL. An addition of 200 μL DMEM containing 1 × 10^5^ cells was to the upper Transwell chamber coated with Matrigel (1:8, Shanghai Yeasen Biological Technology Co., Ltd., Shanghai, China), with 600 μL DMEM containing 20% FBS to the lower chamber. After conventional culture, the fixation of the invaded cells was with 4% paraformaldehyde and stain was with 0.5% crystal violet solution (prepared from methanol). Then, after removing of the cells on the upper surface of the filter, imaging of each filter-fixed cell was in five random fields under an inverted microscope (XDS-800D, Caikon Optical Instrument Co., Ltd., Shanghai, China) and calculation of the number of cells across the membrane was manifested. Implementation of the migration experiment was done without the Matrigel coating [27].

### 2.7 Matrigel tube formation

Coating of Matrigel (0.5 mmol/L) was with pre-cooled 96-well plates. Starvation of HUVECs was serum free, and then re-suspension was in DMEM to produce cell suspension. Next, the seeding of cell suspension (1 × 10^5^ cells/mL) was into a matrix gel coating containing all kinds of A549 cell conditioned medium with setting 3 repeat wells for each treatment. After incubation of the plates, obtaining of photographs was done under the Leica Inverted Phase Contrast Microscope. The counting of the number of intact capillary cavities surrounded by cells was via Image-Pro Plus (version 6.0) at a microscope magnification of 100 times, with a minimum of 3 fields per group [28].

### 2.8 Reverse transcription quantitative polymerase chain reaction (RT-qPCR)

St. Louis, Missouri, USA) as per manufacturer’s instructions. Conduction of reverse transcription was in the S1000 Thermal Cyclist (Bio-Rad) via PrimeScript RT premix (Takara Biomedical Technologies, Beijing, China). The performance of real-time PCR was via the KAPA SYBR FAST qPCR Master Mix kit (KAPA Biosystem, Wilmington, MA, USA) in the CFX96 Real-time System (Bio-Rad). Implementation of RT-qPCR of the samples was performed in a real-time fluorescence quantitative PCR instrument (ABI7500, ABI, Foster City, CA, USA). The presentation of all primers employed in these experiments is shown in Table 1. Application of glyceraldehyde-3-phosphate dehydrogenase (GAPDH) or U6 was as loading controls, with 2^−ΔΔCt^ method [29] for calculation of expression.

**Table 1.** Primer information of fluorescence quantitative PCR

Genes	Forward primers (5ʹ-3ʹ)	Reverse primers (5ʹ-3ʹ)
GAPDH	GGGAGCCAAAAGGGTCAT	GAGTCCTTCCACGATACCAA
let-7d-5p	GGCGAGAGGTAGTAGGTTGC	CGGCCCAGTGTTCAGACTAC
NAP1L1	AGGGACGTGGGACAGTTCGTA	TTTCGAAGTCTGCAGCAAGGATAG
U6	CTCGCTTCGGCAGCACA	AACGCTTCACGAATTTGCGT

### 2.9 Western blot

Lysis of the cells was via Radio-Immunoprecipitation assay lysis buffer (Invitrogen) and determination of protein concentrations was via the bicinchoninic acid protein analysis kit (Pierce, Rockford, IL, USA). Dissolving of the total protein (20 µg) was on 10% gel via sulfate polyacrylamide gel electrophoresis and electroblot was onto Polyvinylidene fluoride membrane (EMD Millipore). After seal with 5% skim milk from Tris-buffered saline with Tween 20, incubation was with primary antibody NAP1L1 (2119, Cell Signaling Technology, 1:1000) and GAPDH (AB8245, Abcam, 1:1000) and horseradish peroxidase conjugated secondary antibody in line with the manufacturer’s protocol. Employment of GAPDH was as a loading control and that of SupreSignal ECL kit (Pierce) was used to detect the bands.

### 2.10 Mouse xenograft model

The gain of the 6-week-old female thymic nude mice was from SLAC Laboratory Animal Co., Ltd. (Changsha, China). Approval of animal handling and testing procedures was via Animal Research Ethics Committee of China–Japan Union Hospital of Jilin University. Subcutaneous seeding of A549 cells was found in the armpits of nude mice at a concentration of 5 × 10^6^ cells/mouse. After the injection, casual assignation of nude mice was into the Huaier and the Control groups, with six mice in each group. The mice in the Huaier were intragastrically given Huaier granule (2.5 g/kg, ig), with that in the control given the same amount of normal saline. Euthanasia of the mice and tumor excision were conducted for immunohistochemical (IHC) analysis [25].

### 2.11 IHC

Detection of vascular density was via CD31 staining. Fixation of the xenograft tumor, embedding in paraffin and section into 4 μm thickness were manifested. After paraffin removal and fluid supplement, seal of the sections and incubation with CD31 antibody (AB28364, Abcam, 1:50) were implemented. Manifestation of quantitative analysis was performed on five randomly selected independent regions of each tumor [30].

### 2.12 The luciferase activity assay

Forecast of the targeted binding sites of let-7d-5p and NAP1L1 was via Starbase database, and wild-type and mutant 3ʹ-untranslated region (UTR) of NAP1L1, named NAP1L1-WT and NAP1L1-MUT, were cloned into pmirGLO plasmid (Beyotime, Shanghai, China) and the construction of mutant reporter plasmid was described as above [31]. Subsequently, co-transfection of NAP1L1-WT/MUT pmirGLO plasmid and let-7d-5p mimic/mimic NC was into A549 cells. Examination of the relative luciferase activity was via a dual luciferase reporter assay system (Dalian, Takara, China) [32].

### 2.13 RNA immunoprecipitation (RIP)

Cells were collected and lysed using a lysis buffer. The supernatant from cell lysates was incubated with human anti-Ago2 antibody (SCBT, Santa Cruz, CA, USA) or negative control antibody (mouse IgG, SCBT, Santa Cruz, CA, USA) for 4 h. Proteins were digested via Proteinase K buffer and the quantitative real-time PCR examined co-precipitated RNAs. Total RNAs were regarded as input control.

### 2.14 Statistical analysis

Processing of all data was via SPSS 21.0 statistical software (SPSS, Inc., Chicago, IL, USA). The expression of the measurement data was as mean ± standard deviation (SD). Paired t-test was employed for comparison of LC and normal tissues, with independent sample *t*-test for that of the other two groups, one-way analysis of variance (ANOVA) for that of multiple groups and Tukey’s post hoc test. Comparison of the data was done between groups at different time points via repeated measurements of ANOVA and Bonferroni post hoc test. Pearson correlation analysis of let-7d-5p and NAP1L1 in clinical samples was manifested. *P* < 0.05 affirmed statistically notable difference.

## 3 Results

### 3.1 *Huaier represses LC angiogenesis and tumor growth* in vivo *and* in vitro

Huaier is clarified to be against on great many tumors. A study has manifested that Huaier can refrain from LC cell advancement [25], but its impact and mechanism on LC angiogenesis are ill-informed. Firstly, after co-incubation of A549 cells with Huaier, detection of the cell advancement was manifested. The treatment of HUVECs was then performed with the corresponding A549 cell conditioned medium for the assessment of angiogenesis. The results affirmed that versus the control, weakened A549 cell progression and angiogenesis capacity was manifested in the Huaier (Figure 1(a–d)). In addition, the same experiment was conducted in H460 cells, and the results were consistent with those in A549 cells (Attached Figure 2(a–d)). For further verification of the repression of Huaier on angiogenesis and tumor growth of LC, gavage of Huaier granule was to the mice with tumor xenograft, manifesting the decline of tumor volume and mass of mice and vascular density in the Huaier versus the Control (Figure 1(e, f)).

### 3.2 Reduced let-7d-5p is in LC; Enhancive one motivates the therapy of Huaier on LC

A study has clarified the under-expression of let-7d-5p in colorectal cancer and repression of the advancement and metastasis [19], while conduction of few studies is in LC. Therefore, the detection of let-7d-5p was done in the clinical samples, clarifying that there was a decline in LC (Figure 2(a)). For further exploration of the impact of let-7d-5p on LC, transfection of let-7d-5p mimic and its NC was into A549 cells treated with Huaier with successful verification (Figure 2(b)). It was manifested versus the Huaier + mimic NC, depressive cell advancement, and angiogenesis ability were in the Huaier + let-7d-5p mimic (Figure 2(c–f)).

### 3.3 Elevated NAP1L1 is in LC; refrained NAP1L1 motivates the therapy of Huaier on LC

A study clarifies the augmentation of NAP1L1 in HCC and the expediting of the growth and metastasis of HCC cells [24], but few studies are manifested in LC. Therefore, the detection of NAP1L1 was in clinical samples, affirming the elevation of LC (Figure 3(a)). For further exploration of the impact of NAP1L1 on LC, transfection of si-NAP1L1 and its NC was into A549 cells introduced with Huaier with successful verification (Figure 3(b)). It was manifested versus the Huaier + si-NC, refrained cell advancement and angiogenesis ability was in the Huaier + si-NAP1L1 (Figure 3(c–f)).

### 3.4 Let-7d-5p negatively modulates NAP1L1

The restrained let-7d-5p and strengthening NAP1L1 were manifested in LC, so a hypothesis was proposed that the targeting was of them. The association analysis of let-7d-5p and NAP1L1 clarified the negative link between them in LC tissues (Figure 4(a)). Subsequently, examination that whether let-7d-5p could control NAP1L1 in A549 cells assured a distinct reduction of NAP1L1 behind elevated let-7d-5p (Figure 4(b)). Next, via forecast of Starbase database a binding site of the two was manifested (Figure 4(c)). Subsequently, further verification of the targeting affirmed that the co-transfection of NAP1L1-WT and let-7d-5p distinctly reduced the luciferase activity of A549 cells, while MUT had no obvious impact (Figure 4(d)). All in all, let-7d-5p negatively modulates NAP1L1.

### 3.5 Elevated NAP1L1 reverses the impact of enhancive let-7d-5p

Next, an exploration of whether augmented NAP1L1 could reverse the impact of elevated let-7d-5p was manifested. Various experimental results affirmed that motivated NAP1L1 enhanced NAP1L1 in A549 cells, facilitating cell advancement (Figure 5(a–d)). Subsequently, an examination of the impact of the NAP1L1 augmentation was performed on angiogenesis, testifying memorable expediting formation of HUVECs tubular structures (Figure 5(e)). Briefly, the elevated NAP1L1 reversed the impact of enhancive let-7d-5p.

## 4 Discussion

LC is a familiar reason for death from solid tumors in the world [33]. Non-small-cell LC takes up nearly 80% of LC diagnoses and comprises disparate subtypes, among which adenocarcinoma and squamous cell carcinoma occupy nearly 40% and 30% of cases separately. A feature of LC is the diversity of genomic transformation, and detection of great many crucial pathological changes is in a great proportion of patients with classification as disease biomarkers [34]. In spite of advances in LC therapy as drug targets, the plasticity of cancer cells clarifies the transience of the response to targeted therapy with stimulating shortdated drug resistance [35]. Hence, it is vital to explore brand-new therapeutic targets for LC.

The effectiveness of TCM has been reported in several disease therapies, like malaria. Huaier is a type of fungus growing on a Huaier tree. In the meantime, approval of Huaier extract is for clinical application via China Food and Drug Administration (No. Z20000109) [36]. Recently, a study of the antitumor impact of Huaier extract was performed in numerous tumors. For instance, Huaier extract restrains of HCC cell advancement via repressing YAP [37]. In breast cancer, Huaier extract enhances the impact of paclitaxel via refraining the NF-κB/IκBα pathway [38]. In the meantime, Huaier curbs melanoma metastasis and angiogenesis [39] and is an efficient antiangiogenic and antitumor agent [2]. In this study, it was proved that Huaier curbed LC angiogenesis and tumor growth *in vivo* and *in vitro*, and was a resultful drug for LC therapy.

MicroRNAs (miRNAs), small non-protein-coding RNAs, are available to negatively mediate gene expression. Identification of thousands of miRNAs is in 25 different organisms via predictive models. Over 1,000 miRNAs are encoded via the human genome with a thorough study of their scope and multiformity. MiRNAs are testified to be linked with the control of numerous basic cellular and physiological processes, like cell development, differentiation and survival [40]. Evidences assure the implication of miRNAs with cancer and the vital impact in cell transformation and carcinogenesis, both as oncogenes and tumor suppressors [41,42]. A study has proposed the reduction of let-7d-5p in ovarian cancer [43]. Additionally, repressive let-7d-5p is in colorectal cancer with depressive advancement and metastasis of colorectal cancer [19]. In this experiment, the measurement and verification of the reduction of let-7d-5p were in LC. Huaier is available to enhance let-7d-5p, which curbed LC cell advancement. The angiogenesis of HUVECs is constrained via the treatment of HUVECs with corresponding cell conditioned medium.

Human Nucleosome assembly protein 1-like (NAP1L) family contains NAP1L1-6 [44]. Versus NAP1L2, 3 and 5 mainly manifested in the brain, NAP1L1 and 4 extensively present in human tissues are greatly conserved [44]. Functions of NAP1L1 protein comprise nucleosome assembly, histone excretion, cell cycle progression, etc. [45]. Detection of NAP1L1 is common in most human tissues and cell lines, but augmented NAP1L1 is frequently manifested in rapidly proliferating cells [44]. Several studies have confirmed the elevation of NAP1L1 in tumors [46,47], clarifying the possible role in human malignancies. For instance, elevation of NAP1L1 is in HCC with motivation of the growth and metastasis of HCC cells [13]. Additionally, enhancement of NAP1L1 is found in pancreatic neuroendocrine tumor metastasis, expediting methylation by controlling p57 (Kip2) for motivated cell proliferation [48]. In this study, NAP1L1, an immediate target of let-7d-5p, was elevated in LC, and enhanced one could antagonize the motivation of elevated let-7d-5p on Huaier’s therapy.

## 6 Conclusion

In brief, Huaier restrains angiogenesis and tumor growth in LC via motivating let-7d-5p and targeted NAP1L1. In this study, more or less update is in the understanding of the mechanisms linked with LC angiogenesis and tumor growth, paving the way for the prevention of LC angiogenesis and tumor growth. It is recommended that more studies are supposed to be conducted to verify the function of the Huaier/let-7d-5p/NAP1L1 axis in LC angiogenesis and tumor growth.

## Highlights

- Reduced let-7d-5p but elevated NAP1L1 were manifested in LC;
- Huaier restrained angiogenesis and tumor growth of LC *in vivo* and *in vitro*;
- Augmented let-7d-5p or declined NAP1L1 motivated the therapy of Huaier on LC;
- Let-7d-5p negatively modulated NAP1L1;
- Elevated NAP1L1 reversed the influence of enhancive let-7d-5p.
